# Digitalization in Disaster Risk Reduction: The Use of Smartphones to Enhance the Safety of Informal Settlements in Iringa, Tanzania

**DOI:** 10.1007/s13753-023-00483-0

**Published:** 2023-04-11

**Authors:** Giulia Jole Sechi, Eefje Hendriks, Maria Pregnolato

**Affiliations:** 1 Department of Architecture, Universitat Internacional de Catalunya, 08018 Barcelona, Spain; 2 Faculty of Geo-Information Science and Earth Observation, University of Twente, 7514 AE Enschede, The Netherlands; 3 Department of Civil Engineering, University of Bristol, Bristol, BS8 1QU UK

**Keywords:** Disaster risk reduction, Housing, Informal settlements, Smartphones, Tanzania

## Abstract

Housing in informal settlements often lacks construction techniques that adopt criteria of resilience to natural hazards. Smartphones are rapidly diffusing in economically developing countries. The aim of this study was to assess the current use of smartphones by the masons of the informal settlements of Iringa, Tanzania, and to identify pathways for improving their construction practices. Data were collected through a mixed method approach that includes in-depth interviews, surveys, and a focus group with masons. The results reveal that only a few masons received formal training, most of them have never interacted with a qualified engineer nor consulted trained professionals when needed. Most masons own a smartphone and they extensively use it to gather technical information from the web, transfer money through mobile payments, share images of construction details, and promote their work on social media. The broad use of smartphones shows potential for enhancing construction quality. This article presents a unique analysis of the use of smartphones in the construction of informal settlements in Tanzania, which could be extended to other countries. Based on the findings, new strategies are proposed to engage with local stakeholders and foster the exchange of technical knowledge for safer settlements via smartphones.

## Introduction

Informal housing is increasingly common, especially in low-income countries (GSDRC 2016; Nassar and Elsayed 2018) but also in lower middle-income countries like Tanzania (Magina et al. 2020). Informal housing lacks the application of engineering principles, as mostly untrained masons cover the roles of “architects” and “engineers,” resulting in extreme vulnerability to natural hazards (Nguluma 2003; Wells 2007). With access to appropriate building techniques, masons could be major agents of change, enhancing resistance of housing to natural hazards (Petal et al. 2008; Chmutina and Rose 2018).

To stimulate their learning process, exploration of effective and innovative communication strategies is necessary. Earlier studies have stressed the importance of the co-production of knowledge and stakeholders’ engagement for learning processes and community resilience (Pasquier et al. 2020; Šakić Trogrlić 2020). Digital tools (e.g., participatory mapping, social media) have shown possibilities for both knowledge exchange and stakeholder engagement (Dave 2017; Kumar et al. 2020). In addition, digital technologies are rapidly and increasingly penetrating daily life in low-income countries (Manyika et al. 2013; Badran 2021).

This study explored how smartphones and related applications can improve the construction quality of informal housing, particularly in hazard-prone communities in Tanzania. We hypothesized that understanding how masons currently use smartphones in the construction process could reveal novel pathways for sharing construction principles that enhance the quality and safety of housing in informal settlements.

The article first reviews the technology currently adopted in developing countries for disaster risk reduction (DRR). Second, we specify our data collection and analysis. Third, we describe how smartphones are used by local masons during housing design, project procurement, and construction in the informal settlements of Iringa in central Tanzania. Finally, we propose strategies to enhance the resilience of informal constructions.

## Background

Digital innovation can play a key role in reducing hazard risks in informal settlements (Kumar et al. 2020). This work is about exploring the potential of digitalization, in particular smartphones, in the construction process for more resilient informal settlements.

### Exploring Digital Innovations to Overcome Current Communication Limitations

Disaster risk reduction strategies have some recurrent limitations. First, some prevention and disaster response programs have a short-term impact, while long-term approaches more efficiently initiate a lasting positive change (Kelman et al. 2011; Hendriks and Opdyke 2021). Second, top-down approaches lack understanding of the communities’ needs, while participative approaches allow contributions of both indigenous knowledge and scientific knowledge (Hendriks and Opdyke 2020). Despite awareness of the benefits of co-production of knowledge, trained architects and engineers continue to use top-down communication methods (Šakić Trogrlić et al. 2021).

The Sendai Framework for Disaster Risk Reduction 2015−2030 (UNISDR 2015) and other studies (e.g., Rahman and Fang 2019; Opdyke et al. 2021) have called for innovative communication strategies that are able to overcome the short-term impact and top-down approach limitations. During the 2016 United Nations International Strategy for Disaster Reduction (UNISDR) Science and Technology Conference, new digital tools such as Ushahidi,^1^ Humanitarian OpenStreetMap Team, and Twitter were praised for their ability to get local communities involved and effectively uncover and share local knowledge (Aitsi-Selmi et al. 2016). However, studies that explore the impact of novel digital tools used by masons to share knowledge for disaster resilience are lacking.

### Necessity to Reduce Hazard Risks in Informal Settlements

**Fig. 1:**
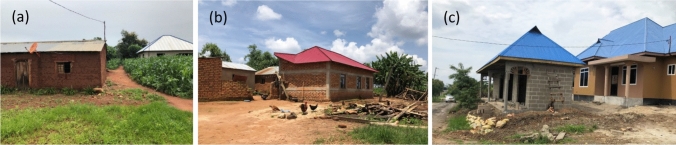
Typical construction techniques in Tanzania. **a** Adobe dwelling; **b** rare burnt clay brick building with reinforced cement concrete (RCC) horizontal and vertical bands; **c** sand block building. Photograph by Giulia Jole Sechi, Iringa, Tanzania 2021

Natural hazard risks are a common threat for informal settlements. The most common risks connected to informal settlements are due to unregulated housing, untrained masons, and unplanned urbanization (Blaikie et al. 2004; CIMA and UNDRR 2019): (1) unsafe location of the buildings (e.g., flood- or landslide-prone areas) (Nassar and Elsayed 2018); (2) variable and unstable connections to services (e.g., water and electricity) (Napier 2000); (3) low-quality building materials and construction details (Nguluma 2003); and (4) susceptibility to natural hazards due to poor construction quality and lack of appropriate building criteria (Lizarralde 2014). The typical construction techniques in Tanzania are rammed earth, adobe (Fig. 1a), *quincha*, burnt brick masonry (Fig. 1b), sand block masonry (Fig. 1c), and reinforced concrete (Edström and Nyman 2017). These construction materials and the structural details lead to buildings that have little ductility and deformability, which negatively affect the buildings’ performance in case of an earthquake, for example.

Improving the robustness of the built environment to withstand natural hazards is pivotal. In Tanzania, there is medium risk of seismic hazard, and high risk of flooding and drought hazards (GFDRR 2020a). The East African Rift System crosses the country, causing considerable seismic and volcanic activities (Chorowicz 2005; Nyblade and Mulibo 2016). In addition, considering the 1979−2018 timeframe, an average of 45,000 people and 4.8 million people per year were affected by floods and droughts, respectively (CIMA and UNDRR 2019).

Masons play a crucial role in the construction and maintenance of informal settlements, as architects, engineers, and registered contractors are usually too expensive for local communities (Nguluma 2003; Makalle et al. 2011). In addition, there is no national structural building code in Tanzania that practitioners can refer to (Lubkowski et al. 2014). Therefore, the lack of quality of the built environment is problematic and even present in the formal construction sector (Kikwasi 2011; Alinaitwe and Ekolu 2014), partly caused by the scarcity of trained workers (Nguluma 2003; Kikwasi and Escalante 2020).

### Rising Digitalization in Tanzania

Smartphone use and broadband Internet are increasing worldwide (Asongu 2015; World Bank Group 2016). In low-income countries, digitalization is penetrating thanks to various factors: (1) mobile payments; (2) the growing interest in big data; (3) the recent Covid-19 pandemic; (4) the diffusion of social media; and (5) e-commerce (World Bank Group 2016; Garg et al. 2021). In 2012, the Internet contribution to the Gross Domestic Product (GDP) in Tanzania (1.3%) was higher than in Russia (0.8%) and Turkey (0.9%) (Manyika et al. 2013). In 2015, the vast majority of smartphone owners in informal settlements in Kenya had Facebook (Wyche 2015). By March 2021, there were more than 29 million Internet users in Tanzania, approximately half of the total population (TCRA 2021).

In Africa, the Internet offers low-cost financial systems for those who previously could not afford bank accounts (World Bank Group 2016). M-Pesa is the main mobile payment system, strongly promoting the diffusion of smartphones and connectivity (Asongu 2015). In June 2009, M-Pesa transferred an average of USD 5.5 million per month in Tanzania (IFC 2010). In the Iringa region, the use of mobile payments increased from 52% in 2013 to 73% in 2017, compared to the 27% (2017) in bank transfers (FinScope Tanzania 2017). These numbers show that mobile payments are playing an important role now and can be expected to play an even more important role in future construction transactions.

### Resilience through Digital Tools in Africa

Digital technologies in low-income countries are already being used to share knowledge and enhance resilience. In Iringa, farmers effectively use mobile phones to exchange agricultural best-practices, for example (Yohakim Nyamba and Mlozi 2012). In West Africa, the United States Agency for International Development and the International Fertiliser Development Centre assisted small farmers from remote communities in digitally connecting with buyers, ensuring fair market prices (USAID and Abt Associates Inc. 2014). Smartphones also proved to aid impaired persons to live in informal settlements in Nairobi (Barbareschi et al. 2020) and assisted women entrepreneurs in Iringa in accessing market information (Kapinga et al. 2017).

Recent studies have also outlined the key role of social media in disaster management and knowledge exchange in Congo (Kavota et al. 2020), as well as in accessing information on sexual health (Pfeiffer et al. 2014) and general health/well-being (EAHRC 2018) in Tanzania. For example, Google funded a project in Tanzania to reach ambulances through smartphones and a Bluetooth emergency dispatch software (Bloomberg Businessweek 2016). Satellite and geospatial big data are playing a major role in early warning systems and relief operations, also through participatory GIS mapping or reporting, such as the mapping of informal settlements in Nairobi, Kenya (Hagen 2011) or flood mapping in Jakarta, Indonesia (Kusumo et al. 2017).

### Growing Use of Smartphones in Informal Construction

Earlier research in the informal settlements of Dar es Salaam (Tanzania) has shown that in 2008 only a few masons were found to use smartphones, primarily to contact clients, and not for accessing technical construction information (Molony 2008). However, at that time the digitalization level was significantly lower. Ten years later, the local government and members of society in Iringa (Msigwa et al. 2018) and Pokhara (Nepal) (Ranabhat and Paudyal 2019) used a mobile application to assess land tenure and facilitate land use planning through participatory mapping. Between 2017 and 2019, mHS CITY LAB created and tested a smartphone application for masons that calculates construction estimates in India (Mehra et al. 2017). In Colombia, Build Change digitalized their housing seismic assessment process and developed a smartphone application that uses artificial intelligence to assess the construction quality (Abello et al. 2020). After the 2021 earthquake, the government of Haiti evaluated almost 180,000 buildings through a smartphone application and provided access to damage and repair reports through a QR code affixed to the houses (Miyamoto International 2022). However, an analysis of the potential of smartphones as a tool to acquire technical knowledge for local masons is missing. Given the high penetration of digitalization into Tanzanian society, this study identified the use of smartphones in the construction process as an underexplored area of research.

## Methodology

This study assessed the current use of smartphones by masons who are responsible of informal settlements. For this purpose, data were collected through a mixed method approach that includes semistructured interviews, surveys, and a focus group. The case study is Iringa (Tanzania). The analysis of the collected data allowed us to identify smartphone-based pathways for improving local construction practices of informal settlements.

### Case Study Selection

This study focused on the district of Iringa in central Tanzania, which is considered an ideal context in which to investigate the potential of digital technologies in the informal construction sector due to the combination of a high penetration of digital technology and extensive informal housing. In Tanzania, informal settlements are common (Ministry of Lands, Housing and Human Settlements Development 2015; Tanzania National Bureau of Statistics 2016). In 2014, 51% of the national population was living in slums (UN-Habitat 2016). In 2017, in the Iringa region, only 5% of the population had formalized their land ownership through a deed (FinScope Tanzania 2017). Furthermore, the annual Tanzanian urban population growth rate was 5.2% (Ministry of Lands, Housing and Human Settlements Development 2015), and uncontrolled urban expansion increases the vulnerability of informal settlements (CIMA and UNDRR 2019).

The region of Iringa is representative of the national Tanzanian average of various parameters, such as the urban/rural population (64/36%), education level (65% primary school, 14% secondary, and 7% tertiary), source of income (40% farmers and fishermen, 21% casual labor, 18% business owners, 12% dependents, 6% formal sector salaried), and wealth distribution (65% of the population in the two lowest quintiles) (FinScope Tanzania 2017). Its capital city, the city of Iringa, had around 151,000 inhabitants in 2012 and it is a typical medium-sized city of Tanzania (Tanzania National Bureau of Statistics 2016), compared to Dar es Salaam (about 7 million in 2021) (Reliefweb 2021). The city of Iringa is characterized by significant smartphone penetration (83% mobile phone owners), higher than the regional (65%) and national (63%) averages; extensive use of mobile payments (86%), higher than the regional (73%) and national (60%) averages; and the Internet network covers 100% of the city (Mawona and Mpogole 2013; Mpogole et al. 2016; FinScope Tanzania 2017).

According to the United States Geological Survey records, the Iringa region experienced an average of one M4.4 earthquake every four years in the last 20 years (USGS 2021). In general, the seismic awareness of local masons is low (Sechi et al. 2022). Considering also the absence of a national building code and the lacking of trained masons (see Sect. 2.1), an adequate seismic structural detailing of the constructions in Iringa is critical. In the Iringa region, the hazard level for river flood and wildfire is high, and for earthquake, landslide, and extreme heat is medium (GFDRR 2020b). Recently, dry periods have become more prolonged, and flooding more frequent (Osiemo and Kweka 2019).

### Data Collection

**Fig. 2:**
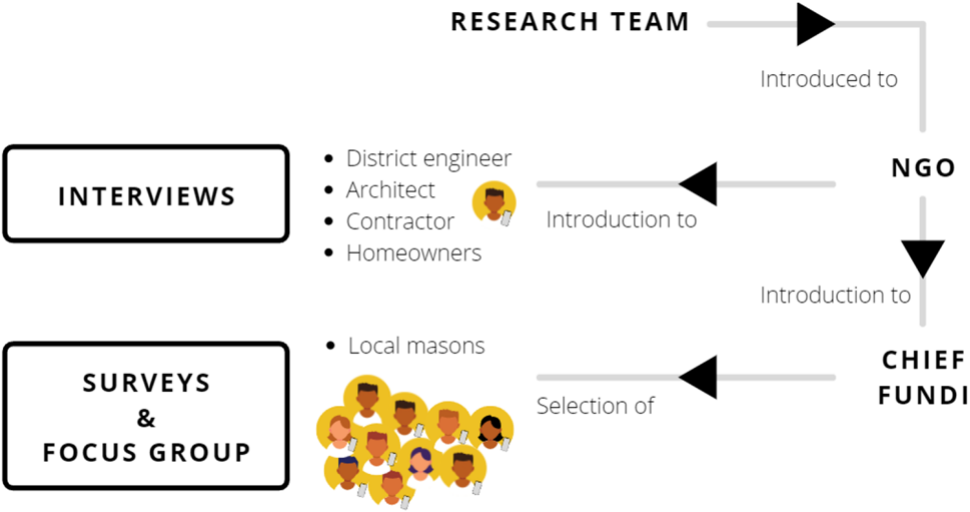
Methodology overview for assessing the use of smartphones in the construction process of homes in informal settlements in Iringa, Tanzania

Data were collected in the period between 24 February 2021 and 3 April 2021. A mixed methods approach was used, including semistructured interviews, surveys, and a focus group discussion (Fig. 2). Due to the Covid-19 pandemic, face-to-face interactions were difficult, restricting the number of participants.

The authors identify themselves as white European women, formally trained in construction engineering, and are intrigued by the use of digital tools to build back safer after disasters. Our positionality may have influenced our understanding, but we have engaged our Tanzanian humanitarian research partner to give meaning to the collected data. The research team consisted of one European researcher, a local guide who also acted as translator and a member of a local nongovernmental organization (NGO). The latter was in contact with the chief *fundi* (*fundi* is “mason” and *mafundi* is “masons” in Swahili) of the wards of Mafifi, Sokoni, Ramadhan, Mfalanyaki, and Mdalagala, which are in the outskirts of the city of Iringa. The chief *fundi* selected 21 masons based on their availability and through the snowball sampling technique (asking the participants to introduce other colleagues). The surveys were performed through KoBoToolbox and were audio-recorded. All interviews were held in English, whereas all the surveys and the focus group discussion were conducted in Swahili. In compliance with the European code of conduct (ALLEA 2017) the scope of our research, our role, the privacy, and the confidentiality of the data collected were discussed with the participants before any interaction. Verbal or written consent were given by the participants prior to participation.

### Semistructured Interviews

The initial objective was to understand whether and how masons use smartphones during informal construction. To pursue this goal, we interviewed five decision makers in the construction process: (1) an architect with more than 30 years of experience, owner of a consulting company; (2) an Iringa district engineer; (3) a manager of a registered contractor company; and (4) two homeowners with construction experience. The questions were open-ended such as: “Who is involved in the construction process?” “What are the usual construction stages?” “How do you relate with the masons?” The architect, the contractor, and the engineer were also asked: “What is the usual training the masons receive?” We also investigated the role of smartphones, asking “whether,” “how,” and “why” they are part of the construction process.

Interviews were thematically analyzed using the constant comparative method (Boeije 2002; Onwuegbuzie et al. 2009): the transcriptions were fragmented, and the fragments were labelled (e.g., design, procurement, construction, smartphone use, training, visual content). For instance, the *design* label has several sub-labels that could be associated to *always*, *often*, *sometimes*, or *never*, such as: use and production of technical drawings and specifications, contact between architect and mason, contact between engineer and mason, use of images to share design ideas, type of app used for sharing. Analyses investigated similarities or oppositions within the same interview and between different interviews.

### Local Masons’ Survey

The findings of the interviews were the basis for the creation of the survey for the participating masons. The scope of the survey was to specify the use of smartphones in the construction process*.* As a result of the sampling strategy, outlined in Sect. 3.2, no particular attention was given to age, gender, and building specialization. For example, out of 21 people only one is a woman, only two people are over 50 years old, and two people are under 29 years old (Table 1). The specializations of the masons cover all the construction aspects, including plumbing and decorations, with 71% of them specialized in concrete works and masonry.

**Table 1:** Demographic details of the 21 mason survey participants in Iringa, Tanzania

Ward name	Number of participants	Gender	Age	Languages	Specialization
Mafifi	1	Male	20−29	Swahili	Concrete, masonry, tiles
	1	Male	20−29	English and Swahili	Concrete, masonry, tiles
	1	Male	30−39	Swahili	Concrete, masonry, paint
	2	Male	30−39	Swahili	Concrete, masonry
	4	Male	30−39	Swahili	Concrete, masonry, tiles
	3	Male	40−49	Swahili	Concrete, masonry, tiles
	1	Male	40−49	Swahili	Timber
	1	Male	40−49	English and Swahili	Concrete, masonry
	1	Male	≥ 50	Swahili	Timber, roofing
	1	Male	≥ 50	Swahili	Plumbing
Mtalagala	1	Male	40−49	Swahili	Concrete, masonry, tiles, paint, timber, roofing, plumbing
	1	Female	40−49	Swahili	Concrete, masonry, paint
Ramadhan	1	Male	40−49	Swahili	Plumbing
Sokoni	1	Male	30−39	Swahili	Paint
Mfalanyaki	1	Male	30−39	Swahili	Timber

The survey consisted of 23 questions. The first section collected general data, such as the ward name, gender, age, specialization, leading to one of the most important questions: “How did you learn your job?” The second part sought to gain a general understanding of the construction process: whether and how masons liaise with trained architects, engineers, or district engineers, whether they usually follow technical drawings, how they learn new techniques, where they look for information when in doubt, what construction aspect or skill they would like to improve. The final section addressed digital technology: “Do you have a smartphone?” and “Do you have regular connectivity to the Internet?” We then explored whether the masons use their smartphone to search for new technical information and whether they would be interested in improving their knowledge via smartphones and relevant applications.

The results were analyzed with the open-source software for descriptive analysis PSPP.^2^ The limited sample size (*N* = 21) did not allow advanced statistical analysis and correlations with confidence (*p* < 0.05) and adequate power (> 80%) (Adam Bujang et al. 2016). In this study, we can only provide indications that point in the direction of correlations that are worth investigating in further research.

### Focus Group Discussion

A focus group discussion with seven local masons was conducted in Iringa on 6 March 2021, after the masons had individually completed the surveys. Open-ended questions were asked by the lead researcher and translated by the local guide. The discussion was first developed around the participants’ work as masons and their internal organization as a group. Then the use of smartphones was discussed, in particular the use of mobile payments. Based on the findings, an additional question to investigate how popular e-payments are was included in the survey in mid-course. Again, the constant comparative method was used: coding and labels that emerged from the analysis of the interviews were used to analyze the focus group discussion. New coding and labels generated by the focus group analysis (such as the *mobile payments*) were used to analyze the interviews again.

## Findings

Our results reflect upon the use of smartphones by local masons in the design, procurement, and construction and training process to identify novel pathways for knowledge sharing that enhance the quality and safety of housing in informal settlements.

### Smartphone Use by Local Masons

The survey revealed that 76% (16 out of 21) of the masons owned a smartphone and they all had regular Internet connection (Table 2). The interviews and focus group highlighted that connectivity can be an issue, but only in remote rural areas. All the masons who did not have a smartphone expressed their desire to own one and were working to achieve that goal.

**Table 2:** Smartphone ownership and social media use for different age groups of masons (*N* = 21) in Iringa, Tanzania

		Smartphone ownership		Social media use to share construction work	
		Number	Percentage (%)	Number	Percentage (%)
Age groups	20−29	1	50	1	100
	30−39	7	78	5	71
	40−49	6	75	3	50
	≥ 50	2	100	0	0

Mobile payments were a major function of smartphone usage: 80% of the masons relied on cash, 60% on mobile payments (M-Pesa), and 10% on bank transfers. The majority of the interviewees and focus group participants commented that mobile payments are extremely common in everyday life and are increasingly used in the construction sector. The owner of the architecture firm claimed that it is not uncommon to pay for an entire new dwelling just with mobile payments.

Of the smartphone owners, 87% use their phones to search for information they do not have. Even individuals who do not own a smartphone seek out information in this way: one participant indicated “I ask my son to look for information on his smartphone,” when he requires new specific technical information. This result is consistent (14 out of 21) with the number of masons who consult websites and social media when seeking construction-related knowledge (Table 2). The use of a smartphone to acquire new knowledge appears to be disconnected from English proficiency, as only two respondents are very basic English speakers (see Table 1). Of the smartphone owners, 81% stated that they would favorably welcome the possibility to use their smartphones to exchange knowledge through external initiatives.

### Role of the Smartphone in the Design Phase

In informal settlements, cooperation with technical experts is rare. Of the 21 masons, 81% *never* interacted with a district engineer and 43% *never* or *rarely* cooperated with an architect or an engineer; 28% of the masons *never* or *rarely* have technical drawings, whereas 57% *sometimes* have.

Typically, masons decide the dimensions and features of the construction together with the homeowners, sketching the plan on paper or reusing an old design. Potential clients usually show photos or snapshots from social media as references, asking the masons to replicate something existing. As a result, smartphones enable a wide exchange of photos and desired design. This approach does not consider any engineering evaluation. One mason claimed that “we improve thanks to homeowners” who show them images and videos of what they would like for their house. In fact, he believes “homeowners are responsible for the design.” One mason mentioned that “I copy from what I see on the streets and from the movies,” while another one said that “I visit other construction sites.” One of the interviewed homeowners shared his experience of building a small cinema. Since the masons were not familiar with that building typology, he shared YouTube videos via WhatsApp (e.g., showing acoustic insulation types and installation techniques).

### Role of the Smartphone in the Procurement Phase

**Fig. 3:**
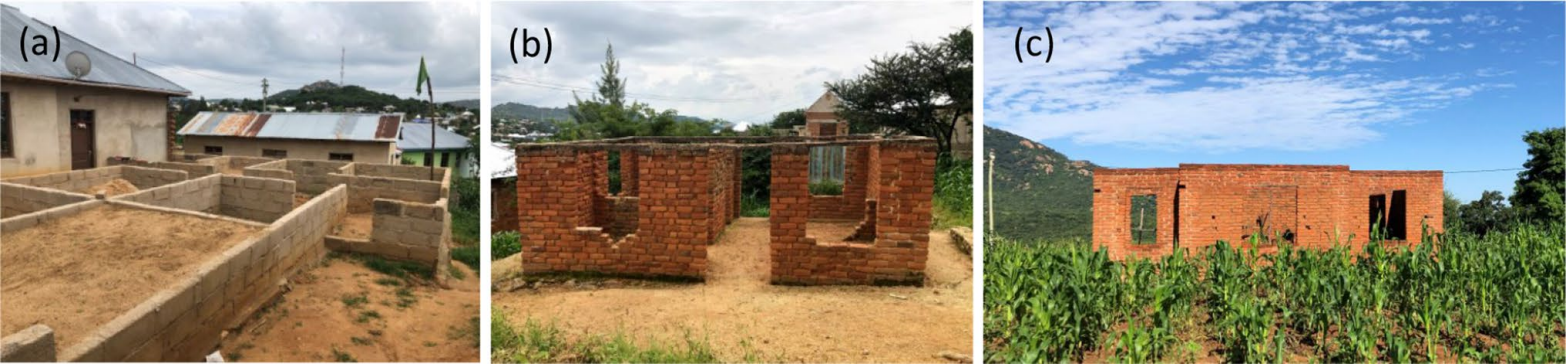
Incomplete sand block (**a**) and burnt brick (**b** and **c**) masonry buildings. Photograph by Giulia Jole Sechi, Iringa, Tanzania 2021

Procurement refers to the practice of obtaining goods and services in relation to the construction. The interviews and the focus group revealed that banks seldom provide micro-credit to households and financial limitations are an issue. Construction is usually started as soon as there is a small budget available; as a result, a large number of buildings stand incomplete and exposed to the weather for considerable time (Fig. 3), jeopardizing the final quality of the house.

Each *fundi* is specialized in a different trade; thus, homeowners liaise with several masons. Of the 21 masons, 76% believe their estimations are well-assessed. Nonetheless, the interviewed stakeholders claimed that the pricings coming from the masons are generally variable and unreliable. Furthermore, during the focus group discussion some of the masons reported that it is challenging to request extra money from the homeowners, and it is common to reduce the quality of the materials to not exceed the budget during the construction.

In this context, “word of mouth” is the most common procurement strategy, followed by social networks: 43% of the masons advertise their work on Facebook or Instagram (see Table 2), using photos that include their contact details. One mason confirmed that the large majority of his clients were acquired through Facebook and “I received requests even from outside my region.”

### Role of Smartphones in the Construction and Training Process

The city of Iringa is home to the Ifunda Technical School and the Vocational Training Centre, which are managed by the Vocational and Education Training Authority (Redecker et al. 2000). The interviewees and the focus group reported that they are inaccessible to most of the masons, due to their cost. The survey results indicate that 81% of the *mafundi* did not receive any formal training but learned from other masons (67%) or a family member (14%). They usually approach the construction sector as *helpers* and then slowly gain independency. The Internet is not a source of knowledge in the first phase (0%), while NGOs are (19%), but they do not retain the same role for follow-up questions (0%).

When masons search for new technical information, the most traditional method (“ask other *mafundi*”*)* and the most innovative one (“consult the Internet”) showed the same attractiveness (67%); 29% look on social media (mainly Facebook, Instagram, and Pinterest) and only 19% try to contact experts (Table 3). Spontaneous utilization of smartphones in the construction process is observable, although there are no specific applications designed to provide information to the masons. Only two participants over the age of 40 mentioned paper-based guidelines, whereas the consultation of the Internet is well distributed across all ages, and only masons over 50 have no interest in social media (Table 2).

**Table 3:** Knowledge sources for learning and consultation used by masons (*N* = 21) in Iringa, Tanzania

	Original knowledge source to learn about construction (How did you learn your job?)		Knowledge source consulted for information about construction techniques (If you need information on construction techniques, where do you look?)	
	Number	Percentage (%)	Number	Percentage (%)
Other masons	14	67	14	67
NGOs	4	19	0	0
Family member	3	14	0	0
Contractor company	3	14	0	0
School training	3	14	0	0
Religious mission	1	5	0	0
Internet	0	0	14	67
Social media	0	0	6	29
TV, radio, or newspapers	0	0	5	24
Experts (contractors or engineers)	0	0	4	19
Paper-based guidelines	0	0	2	10

Social media, websites, and construction sites managed by registered contractors are a source of information especially for “borrowing” aesthetic features. Visual content seems to appeal the most. When asked, the masons indicated that written text found on websites is too complex, while images and videos are more immediate and entertaining. The TV program Ujenzi, which was created by the government to train masons, was mentioned as a source of new knowledge by 3 (14%) of the masons. This result is aligned with the latest governmental DRR strategies, whose efforts are focused on launching a new radio and TV program to raise awareness of natural hazards (The United Republic of Tanzania 2021).

Almost all participants (90%) expressed a desire to be more knowledgeable in a certain field. Remarkably, almost 25% of the respondents would like to be more knowledgeable on *Tall* or *modern buildings*. Only one person out of 21 mentioned *Safe structural details*; 33% indicated *Good toilet facilities*, 24% *Water tightness for heavy rain*, and 14% *Good light and air circulation*. None of the natural hazards that affect the area (Sect. 3.1) were mentioned. This result aligns with Mehra et al. (2017), who claim that households are predominantly influenced by aesthetic/design criteria and societal standards during construction.

## Discussion and Recommendations

The results highlighted three main aspects: (1) there is a widespread use of social media and smartphones; (2) visual content is more attractive than written text; and (3) mobile payments are commonly used. This section is a reflection on these findings and proposes two initiatives to improve knowledge and self-training among masons, through channels they are already familiar with.

### Co-benefits of the Use of Smartphones for Construction

Digital applications are adaptable and functional for facilitating multidisciplinary and multi-hazard approaches. Images proved to be significantly used and exchanged to show the desired design and to promote the masons’ work on social media. Virtual visual content, as opposed to paper-based guidelines, can be rapidly modified (e.g., to adapt to new building codes) in the exchange of designs and techniques between user and constructor, creating opportunities for co-construction. This exchange creates opportunities to select appropriate techniques that account for contextual and financial limitations in informal settlements. Although physical presence and face-to-face discussions are valuable and irreplaceable, the flexibility of remote online tools has been proven successful, for instance, during the Covid-19 pandemic (Kumar et al. 2020; Garg et al. 2021).

We also recognize the potential of a digital approach to overcoming biases. Vulnerable groups (e.g., inhabitants of informal settlements, women, or ethnic minorities) could anonymously consult and share information with people who would otherwise be inaccessible (Kapinga et al. 2017; Wyche and Olson 2018; Barbareschi et al. 2020). Therefore, it would be beneficial to explore different digital communication solutions that engage different groups of societal actors and vulnerable groups in construction decisions. Apart from masons and engineers, solutions could target decision makers and policymakers, local authorities, communities, and educational institutions. An adequate building code is currently absent (Sechi et al. 2022) and multi-stakeholder digital approaches may raise awareness of the importance of national standards, facilitating application and supporting a new generation of (informally) trained practitioners. A collective safety culture with regard to natural hazards and the built environment could be fostered starting with students and in community centers, where digital tools can be used as learning aids.

### Virtual Digital Hub to Connect Masons and Engineers

Existing web-based platforms (e.g., PreventionWeb and the World Housing Encyclopedia) are valuable sources of knowledge for engineers, but are too complex for untrained masons and not translated into local languages (e.g., Swahili). As a result, it appears that both engineers and masons use the web to exchange technical knowledge, but in different virtual spaces. Furthermore, there is potential for engineers to be more involved in currently informal construction processes, as the results show that only 19% of the masons refer to engineers or trained contractors when they have technical doubts.

A smartphone-based initiative could include ad hoc social media groups that connect various stakeholders and host discussions on specific topics (e.g., foundations), in a specific geographical area (e.g., Iringa). A Facebook group could bring together government experts (e.g., a district engineer who moderates the group), academic experts (e.g., a PhD student), masons, and households. Masons would have the opportunity to directly engage with the other members of the community. The NGOs could also participate to better understand the local context or to share their experience in the field. The outcome could be the co-creation of construction details or the sharing of best practices.

This co-production of knowledge with multiple stakeholders is an example of a participative long-term approach, as opposed to top-down approaches that are disconnected from the context and do not establish lasting relationships. Co-creation of houses fits better with informal construction processes where households self-construct or collaborate with construction workers.

Knowledge exchange through virtual hubs should aim to verify structural safety of shared technical solutions. Since there is currently no building code that accounts for seismic risks, it is likely that unsafe solutions will be shared. We recommend governmental bodies to invest in the development of a structurally sound building code and the creation of accessible visual content to explain key messages for housing safety. Based on governmental indications, disaster risk reduction elements can be introduced by the moderator, to raise awareness and improve the resilience to natural hazards. A similar initiative could be aligned with the strategies of the Tanzanian government, which is engaged in a campaign to raise awareness and increase the resilience of the built environment (The United Republic of Tanzania 2021).

### Technical Training through Mobile Payments

**Fig. 4:**
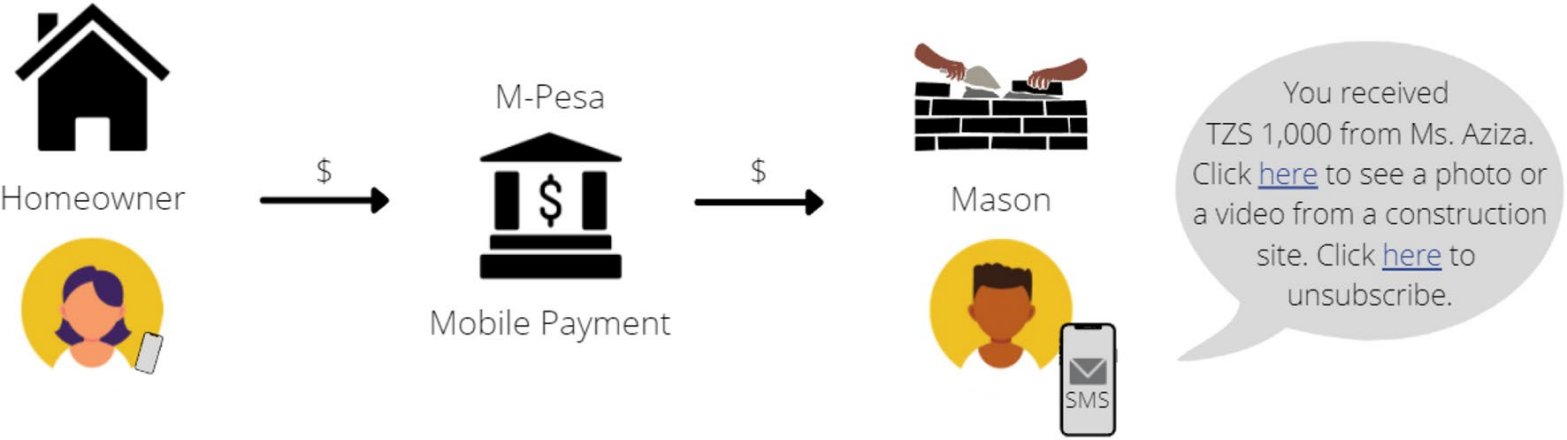
A link can be added to the message that is received by the mason for a transaction related to construction

Considering the significant use of mobile payments by the masons in informal settlements, a partnership between the government and Vodacom (owner of M-Pesa, the major mobile payments’ company in Tanzania) could be established to allow the *mafundi* to receive “extra information” every time they receive money associated with construction work (Fig. 4). Every time a money transaction is completed the parties involved already receive a message from the mobile company confirming the operation has been successfully executed. Direct links could be included to reliable visual content of technical knowledge (videos or images), or to the social media groups discussed in Sect. 5.2. Videos could be taken from the local TV program Ujenzi that has been created to inform and train masons on various construction techniques (see Sect. 4.4). This approach is mainly top-down, thus particular attention should be given to privacy and permission: for example, the masons shall opt in or out of the special free service at any time, and indicate which topics they would like to know more about (e.g., wall connections).

### Challenges and Future Research

One mason out of four did not have a smartphone. The digital divide is an inherent limitation for digital approaches (Shaw 2020). A strategy based on virtual connectivity could aggravate the isolation for those who do not have access to digital tools (Kelman 2020). Furthermore, despite the extensive use of the Internet, the masons did not raise any concern about the quality of the information they can find on the web or about their privacy. Future studies could investigate the so-called “African digital rising” from a construction perspective, advancing mitigation strategies for those who may be excluded by the solutions proposed, analyzing the ethical and information quality aspects, and exploring other countries where digital penetration is significant. As a result, the local capacity building strategies should not exclusively rely on initiatives that use digital tools, because not digitally connected households also need to be reached.

Women were found to be underrepresented in the local masons’ community and only one person in our sample was female. Demographic data from Iringa (Tanzania National Bureau of Statistics 2016) shows that smartphone ownership is lower for women-led households (48.6%) than for men-led households (66.5%), and therefore women could be less engaged in digital-based strategies. However, a broader and more statistically complete survey sampling will be required in the continuation of this line of research.

Our suggested interventions are based on our limited exploratory data collection in one region. The impact of these interventions on overall housing safety in Tanzania should be verified in further research, including samples of structural safety assessments across multiple districts.

## Conclusions

Our research investigated how smartphones are used by the masons of Iringa, to identify innovative strategies that could improve the quality and safety of housing in informal settlements.

We found that most of the masons own a smartphone, and its use is variably integrated into all stages of the construction process. Masons consult the Internet to acquire new technical knowledge, advertise their work on social media, and use mobile payments to be paid for their services. Formal training is expensive, and therefore informal training is gained through websites and social media, especially through visual content. Design ideas and construction details are shared with no supervision by an engineer; quality and resilience are neglected, favoring aesthetic criteria and cost reduction. Most of the masons indicated that they would positively welcome the possibility of using their smartphones to access new technical knowledge through external initiatives.

This exploratory study highlighted that smartphones are already playing a prominent role in technical knowledge sharing in Tanzania. Our proposition is that new disaster risk reduction strategies for informal housing can capitalize on the current use of social media and mobile payments to improve masons’ technical knowledge. For example, social media can host digital groups where engineers, architects, and masons connect to share construction details and principles from building codes. Furthermore, masons can receive ad hoc training in the form of images or short videos through the mobile payment system. Future research could explore these proposals in more detail.

## Footnotes

1 A platform to mobilize the communities and gather data: https://www.ushahidi.com/.

2 https://www.gnu.org/software/pspp/
